# Addressing Marginality and Exclusion: The Resettlement Experiences of War-Affected Young People in Quebec, Canada

**DOI:** 10.3390/children6020018

**Published:** 2019-01-30

**Authors:** Andie (Saša) Buccitelli, Myriam Denov

**Affiliations:** 1 Social Service Program, Dawson College, Montreal, QC H3Z 1A4, Canada; 2 School of Social Work, McGill University, Montreal, QC H3A 2A7, Canada

**Keywords:** war, migration, resettlement, refugee, youth, exclusion, cultural norms, Quebec, Canada

## Abstract

Accessing meaningful forms of support can be an onerous experience for young people resettling from war-affected contexts. In addition to facing linguistic and financial barriers in this process, these young people negotiate care systems that are often structurally and culturally insensitive to their unique needs, values, beliefs, and intersectional experiences of oppression. Drawing on interviews with 22 young people from war-affected areas living in Quebec, Canada, this paper critically examines how dominant cultural norms and social relations in Quebec’s health, social and educational services network shape their experiences in seeking care, healing and belonging. Alternative care systems and approaches, as proposed by the participants, are then explored. The findings emphasize the need for spaces and care services where war-affected young people’s identities and lived realities are validated and represented.

## 1. Introduction

Wars continue to alter the lives of children, young people, and families around the world with devastating social, economic, political and psychological effects. More than 600 million, or one in four, of the world’s youth, live in areas affected by armed conflict and insecurity [1]. Within these wartime contexts, children and young people are killed, exploited, abused, injured, orphaned, separated from family and/or recruited into armed groups (for the purpose of this study, “child” is defined as any person under the age of 18 years, in accordance with the United Nations *Convention on the Rights of the Child* (UNCRC), while “young person” is defined as anyone between 15 and 30 years of age).

Countries like Canada are indirectly, yet intimately touched by war. Each year, thousands of children enter Canada, fleeing war zones [2]. Refugees admitted to Canada represent approximately 10% of the roughly 285,000 new permanent residents in the country every year [3]. In 2016, the United Nations High Commissioner for Refugees (UNHCR) lauded Canada’s resettlement of 46,700 refugees, a record high in refugees admitted since the implementation of the 1976 Immigration Act [4]. This represents a 133% increase compared to the previous year. Additionally, nearly half (47%) of refugees admitted to Canada in 2016 were children [5]. In the year of 2017, the Government of Canada reported a total of 50,380 asylum claimants (processed across the country (it is worth noting the shift in language, using ‘asylum claims’ instead of the terms ‘refugee claim’/ ‘refugee claimant’ as used in the Immigration and Refugee Protection Act). Based on recent figures reported by Immigration, Refugees and Citizenship Canada (IRCC), as of 29 January 2017, Canada resettled a total of 40,081 Syrian refugees [5]. This is due in part to Canada’s humanitarian transfer of Syrian refugees with UNHCR’s support, as part of the Canadian government’s explicit commitment to resettle refugees with a “renewed focus on reuniting families” [5]. The government has also emphasized the importance of combined efforts with civil society and service providers to support refugees’ resettlement and integration [4].

Resettlement from a war-affected context is both complex and multifaceted. Young people displaced from war zones witness or directly experience severe and unimaginable violence and upheaval. For those who make their way to Canada, war-related mental health distress may occur alongside poverty, discrimination, isolation, language barriers and difficulties in school [6,7]. Studies on young people with refugee backgrounds in Australia point to similar resettlement realities [8,9,10,11,12,13,14,15,16,17]. With growing numbers of asylum claimants and their families receiving permanent residency—and becoming interwoven in the Canadian social fabric—it is critical that psychosocial programs and interventions address their needs, as individuals, families and communities. Moreover, there is a greater need for culturally responsive practice with children and families from war-affected and refugee backgrounds that accounts for the diversity and heterogeneity of their needs and experiences.

The province of Quebec has a unique independence with regard to immigration policies since the signing of the Canada-Quebec accord in 1994. This accord allows the province to develop its own criteria for selecting immigrants and refugees from abroad. However, under this accord, Canada remains in charge of the selection and processing of asylum seekers. The accord was negotiated in order to ensure the promotion of the French language and the uniqueness of Quebec’s cultural identity. It also set Quebec apart from Canada by its use of an intercultural model rather than a multicultural model within the policy [18].

There is an emerging literature on the experiences of young people with refugee backgrounds and from war-affected areas in multiple contexts around the world [8,9,10,11,12,13,14,15,16,17], including Canada [7,19,20,21,22,23]. However, the lived realities, resettlement experiences and service provision needs of this population of children and young people deserve greater attention and ensuing action. In particular, how do young people from war-affected countries living in Quebec negotiate resettlement? How and where do these young people seek support? How do young people experience support in more formal spaces, including those integrated as part of Quebec’s health, social services, and education systems? Answering these questions is key to informing and improving resettlement programs and services.

In exploring these experiences, this paper adopts the theoretical framework of intersectionality. Intersectionality posits that people’s realities are shaped by the interaction of multiple forms of oppression (e.g., racism, sexism, ableism and classism), creating entirely new and unique experiences of marginalization [24,25,26]. Moreover, intersectionality supports an understanding of individuals’ identities and experiences of oppression as fluid and mutable [27]. This is integral when exploring the complexities of individuals’ social locations in light of migration and the shifting experience of oppression when accessing different spaces. This perspective is also useful in considering how historical processes of oppression impact the spaces young people navigate in the present (e.g., colonialism) [27]. In adopting an intersectional framework, this paper will critically explore how different forms of structural oppression, including insecurity, linguistic discrimination, racism, status and assimilation, shape war-affected young people’s wellbeing and experiences in accessing support in different settings. Specifically, the paper aims to consider how dominant cultural paradigms and social relations in different systems and spaces affect young people’s experiences of healing, support and exclusion.

## 2. Methodology

To examine the resettlement experiences of young people affected by war living in Quebec, in-depth interviews were conducted with 22 young people (11 male and 11 female) living in Montreal and St-Hyacinthe, a small-sized city located approximately 60 km east of Montreal (ethical approval number: #116-0912). These two contexts were chosen as they provide a glimpse into an urban setting and a more rural, or semi-rural, setting. Moreover, both contexts had relatively large numbers of war-affected young people with refugee backgrounds. The young people were recruited through the assistance of local settlement workers, and social workers. In addition, after interviewing several young people, we relied on snowball sampling to reach other potential participants. Participants were between the ages of 15 and 30 at the time of the interview/focus group and had been living in Canada from 6 months to 12 years. Their countries of origin included Colombia, Democratic Republic of Congo, Nepal, Rwanda, Sierra Leone, Sri Lanka, Togo and Zimbabwe and were conducted over a 10-month period, beginning in January 2013. Interviews were carried out in French, English or Spanish, depending upon participants’ language preference. All interviews were audio-recorded with permission and subsequently transcribed.

The interviews were conducted by the second author, alongside a team of researchers involved in the project. Interviews took place at community centres or in the office of the researchers and lasted between one and two hours. Given our desire to ensure participants’ overall safety and security during the research process, we aimed to include young people who were in some way along the “healing” journey. In this sense, participants were perceived as “leaders” by local social workers and settlement workers, who assisted in participant outreach. Nonetheless, to ensure the safety and security of participants, support systems—in the form of ongoing support and interventions from the settlement workers and social workers who assisted with participant outreach—were put into place in the aftermath of interviews.

To ensure that the participants’ perspectives were appropriately captured, six young people from war-affected areas (aged 17–25) acted as ‘youth advisors’ during the design phase of the research. These young people were all from Colombia, having sought refugee status in Canada as a result of the armed conflict, and had been living in Canada between 2 and 5 years. A preliminary group discussion, conducted by the second author, was held with these six young people that outlined the goals and objectives of the research and explained our desire to develop interview questions that would be relevant to pose to potential research participants. The youth advisors were then asked to participate in a group discussion that addressed the opportunities and challenges that they themselves had experienced when resettling in Canada. In addition, the youth advisors worked individually and collectively to devise sample interview questions that they felt would be important to ask young people from war-affected areas who recently resettled to Canada. Youth advisors also offered critical feedback on our overall research objectives and provided direction in terms of appropriate methodologies emphasizing the importance that the interviewers “be present and ready to listen.” A key contribution of the youth advisors was their emphasis on the importance of talking about young people’s wartime pasts, even if the project’s focus was on the issue of resettlement. As one of the youth advisors explained:

It is important to know the circumstances of youth prior to their arrival in order to understand what they may need. Some youth may seem to be functioning and well on the outside but on the inside, they may have a lot of repressed emotions and feeling. Experiences lived [during war] are not forgotten and are therefore expressed in different ways.

The interview protocol developed based on their feedback, explored young people’s wartime lives and experiences in the context of their countries of origin, the impact and legacy of the war on their lives, both past and present, as well as the realities of flight and resettlement to Quebec, Canada. The interviews also explored participants’ sources of support once in Canada, both informal and formal, including their experiences with social and health services, as well as their comfort levels in sharing their stories of war, migration, and resettlement (For the purpose of this paper, “formal” spaces include organisations and agencies that are integrated into the Quebec government’s publicly-funded education, health and social service systems. Examples include hospitals, schools, public clinics, such as CLSCs (*centre local de services communautaires*), and youth protection agencies). In addition to interviews, two focus groups were conducted with the same young people who had been interviewed as part of the research project. These focus groups, conducted by the second author and two members of her research team, aimed to trace the diverse opportunities and challenges that they faced upon resettling.

Data were analysed using a grounded theory approach, whereby through inductive analysis of the data, researchers gained an understanding of the patterns that exist in the social world under study, that are firmly grounded in the experiences of the individuals acting in it [28]. To facilitate data analysis, a conceptual coding tree was created with the assistance of HyperResearch—a qualitative analysis software. Through ongoing data analysis, participants’ experiences of both support and exclusion in formal and informal spaces emerged consistently in the young people’s narratives. These themes are addressed in greater detail in the following section.

## 3. Navigating Resettlement: Experiences in “Formal” Spaces

*People sometimes think you are so traumatized. But what makes it more traumatizing is the system. You understand what I’m saying? It’s the system.*

—David, participant.

The experiences of war, flight and resettlement have complex implications for people’s social, emotional and mental state of being. Loneliness, isolation, feeling uprooted, uncertainty about the future and a generalised sense of insecurity and fear featured prominently in participants’ stories. After settling in Quebec, these young people were also expected to negotiate their way around a variety of systems whose inherent norms are often experienced as unfamiliar and disconcerting. Quebec’s education and health and social services systems play an important role in many of these young people’s post-resettlement lives. In the province of Quebec most, if not all young people, must attend “*des classes d’acceuil*” (‘welcome classes’) and begin the process of “*francisation*” in order to learn French and become better “accustomed” to local values, culture and social relations [29,30].

Moreover, many young people coming from war-affected areas may be referred to health and social services in order to receive psychosocial support for mental-health issues related to war, displacement, flight and resettlement. However, research has shown that minorities and immigrant families in Canada significantly underutilize mental health services and instead seek support from community organizations [31,32]. What is the reason behind this?

In many ways, schools, clinics, such as CLSCs, and other public services offered provincially in Canada are meant to support opportunities for learning and healing. Therefore, referring, or requiring young people to access these spaces seems reasonable given their past experiences of violence and the challenges that come with negotiating a new sociocultural environment. Although these spaces may act as centres of healing, learning and support, to varying degrees, they may simultaneously be experienced as sites of racial oppression, and assimilation. Upon entering these spaces, participants often reported confronting norms, values and social relations that did not speak to their unique realities, invalidated their beliefs, and eclipsed their sense of identity.

### 3.1. Health and Social Services: Conflicting Paradigms of Wellness

Canada’s health and social services systems have been said to be heavily rooted in a modernist, Western biomedical understanding of health and illness. The focus is largely on the individual and the use of scientific tools and methods to assess and address the manifestation of disease [33]. Treatment often involves “separating” a person deemed “mentally ill” from the rest of society, sometimes through the use of labels (i.e., diagnoses) and/or their literal segregation from others (e.g., institutionalization) [34].

In interacting with the health and social services system, many young people in our study were faced with this Western conceptualisation of mental health. A common presumption is that most of these young people have been traumatised by the experience of war, and stand to benefit from a psychosocial assessment, followed by some form of treatment (i.e., therapy and/or medication). Similar processes of medicalisation, whereby people from refugee backgrounds are treated as “traumatised” and “psychologically harmed”, have been highlighted in studies exploring resettlement experiences in the Australian context [12,15]. Of course, these assumptions, along with their associated theories and diagnoses, stem from a particular sociocultural standpoint: The notion of “mental health” was developed in North America and Europe [34]. As this participant points out:

The system is very, very culturally biased. They are very ignorant. Because for them it’s just—they have this theory […]. People come here from a traumatized country and they became more traumatized now […] Western theory—applying […] Western theory, it doesn’t apply to some of us.

*(David)*

Refugees affected by war are readily screened for Post-traumatic Stress Disorder (PTSD) [15,35,36]. Although it may be the case that many people affected by war exhibit PTSD-like symptoms, it does not necessarily mean that some form of psychosocial intervention is required or even desired [15]. In fact, many young people in the study did not feel any particular need to access mental health support and described how seeking “outside” support was a peculiar concept for them:

*It’s not like I come from a country where [formal mental health support] is in abundance, because it’s not. They don’t have sort of a psychosocial worker […] Granted, the family unit from those countries is so strong that there is never ever a need to start going outside—seeking help outside*.

*(Andy)*

*I never saw a psychologist. I never saw a therapist. But I made it on my own mind, created my own survival until I made it*.

*(David)*

I don’t think someone can help me […] Because I think what I feel is normal, no? What I mean by normal, it’s like—what I feel is—it’s supposed to happen.

*(Jasmine)*

Many participants did not view their emotional and mental state as something that required professional attention. Finding power in oneself to heal, and looking for support from family and loved ones, appeared to be more organic avenues for the participants. The last quote reflects a perspective of mental health that differs from the biomedical model: rather than viewing how a person feels after a traumatic experience as an issue, it is important to understand that these emotions are perfectly “normal” in light of the difficult circumstances they faced in the context of war and violence.

Other participants who had considered to use, or who had actually accessed formal mental health resources, shared their reflections about the impact and consequences of these services:

Okay, yes. So I arrive, I take another appointment and I cry and cry. Ah! Okay!, I said: “Finally, it’s exhausting, this system! I’m never coming back... So, I told myself I should go see the psychologist—because me—maybe I had a preconceived idea of a psychologist who would tell me, who would listen to me and tell me: “Pa-pa-pa-pa! You are cured!” But then—after I asked myself: “What is illness?

*(Naomi)*

The following participant reflected upon whether a practitioner from Canada would be able to understand the context of the war in which she grew up:

And it’s the institutions that are not ready, or are not specified. They are for everyone, for all people in the school. So...but now, if I go see a psychologist, I tell myself: “Are they really going to understand my situation ? […] Are they really ready to listen to me?”

*(Anna)*

These testimonies highlight how some formal spaces may be unprepared and ill-equipped to support these young people. Moreover, are these professionals engaging and critically considering the structural realities of racism and xenophobia and how they may impact upon their everyday practice, as well as the everyday lives of their clients? As this participant astutely states:

*[One] of my criticisms of social work is—I’m going to be straightforward, here. It’s whites, educated […] How many people do you find in social work that are a minority? Maybe there are one or two. But how can you understand someone’s culture if you have just studied it from you own perspective? […] Refugees come here, all they want is the support to have safety. But, no, they don’t feel safe. Because the same people they’re talking to are the same person who is in the system. Social workers here, one of the greatest biases they have is that they don’t see it from the client’s own view. They see it from their own theories*.

*(David)*

*[The] people who work in the system have to be trained to help cultural education. Because a lot of people that I have spoken to find these people are culturally ignorant. If you walk into a clinic and you cover your head, already they have some perception about you. If I walk into the clinic being black or whatever, I don’t know, people have shared that with me*.

*(David)*

In this sense, in order for people to benefit from formal spaces, specifically those that are part of the nation’s public system, it seems imperative that these spaces are experienced as secure, validating and relevant.

### 3.2. Education: Feelings of Exclusion and Marginalisation

The participants also encountered a variety of challenges in navigating educational institutions. Here too, many were faced with social relations, norms and rules that rendered them feeling “othered.” This participant, who was forced to abandon his schooling for several years, due to the break out of the war in his country of origin, explained how he was discouraged from continuing with his high school education once settled in Canada:

*They told me that I would never go to university. And I said to them, “Why?” They said because you’re 22 now and here you have to go through adult [high school] system. Well, I’ll apply to [name of university] and tell them the reason why they should accept me. I wrote them a letter of intent on my own and sent it out to them. They accepted me*.

*(David)*

I was traumatized about coming [to university]. Psychologically, I was traumatized […] once, I finished this paper. I handed in. [...] The professor called me and said […] to me “Are you the one who wrote this paper?” I said, “Yes, I studied the book and I wrote it.” She said, “Because we have some doubts. Your colleagues are thinking somebody gave it to you.” I said, “You see? Why would you question the fact that I wrote this? I’ve been here—this is my final year.” I was so upset. I brought all the papers that I have written. She apologized. But I said, “You want to discourage me to drop out of University. Then you will put me in statistics as one African, or whatever immigrant population, this, this, this.” And she apologized. And I was sitting like this, and she said, “I’m sorry.” And I cried in her office.

*(David)*

*The system equals racism. It includes [name of university], includes [name of university], includes the hospitals*.

*(David)*

Because it’s obvious that at school, they see us like an immigrant. It’s there that I feel the difference between being an immigrant and you as a person, what you [experienced/lived through]. They see you as an immigrant, they always ask you the same question: “where do you come from?” always this. And they don’t go much further than this. It doesn’t go much further. Like… I don’t know, I have Quebecois friends and they don’t go further than that. Because they change the subject, talk about something else. Then they don’t get to know you beyond that. They don’t feel attached to you. You don’t get enough of a connection.

*(Anna)*

Similar to health and social service contexts, educational institutions were often experienced by participants as sites of surveillance and interrogation. Intersecting prejudices of age, race and status were experienced in ways that the participants described as devaluing their desires and erasing their personhood. In both cases, the young people disregarded and pushed back against attitudes and structures that they experienced as exclusionary and oppressive. Nevertheless, it is important to recognize that the culmination of these oppressive experiences have profound implications for people’s psychological wellbeing and sense of trust and comfort when it comes to Quebec’s education, health and social service systems.

Anna’s quote above sheds light onto the often-reductive constructions young people with migrant backgrounds face in relation to their identity and experience. Anna described how such categorical understandings rendered her feeling as though people do not wish to see past this part of her reality and experience, and, as a result, she did not feel connected to some of her peers. In other words, the images many Canadians have of what constitutes an “immigrant” places constraint on the development of intimacy and genuine connection.

Furthermore, in the province of Quebec, participants also described experiences where they were met with impatience and sometimes mockery when trying to learn and speak the local French language:

Yes, in the school, it’s going good, well. So, teachers are also nice, and students also, but sometimes, I don’t know—most persons are getting mean when I speak in French, so they’re going to do laughing, and I feel very sadness.

*(Saddya)*

Yeah, because I work on phones [as a telemarketer, part-time]. Like, sometimes, you talk with someone, they say: “You have an accent”….Yeah, I understand, we have an accent, but they also have an accent. Like, they don’t hear what you’re saying, so they need another person. But there is a way, maybe, to say it politely. But last time, I got a customer, and she was like: “Ha-ha, you’re funny”. I’m like “Why?” “Why? You are funny. You have an accent and I can’t understand you”. She was like: “Next time, and make sure you write in your notes there. Next time, when you call, you have to ask a Quebecois [a native or inhabitant of Quebec, typically one who is French Canadian] to call me. I don’t want any other person to call me, apart from a Quebecois.”

*(Jasmine)*

While these quotes highlight the primacy of the French language in various educational and employment settings in the context of the province of Quebec, they also illustrate how participants were regularly confronted with experiences of exclusion, discrimination and racism. The challenges that come with learning a new language, in a completely new context, are compounded by the negative, often shaming, reactions many young people experience at the hands of their peers when attempting to learn and speak the language.

Specifically, most, if not all, young people resettling in the province of Quebec are placed in an intensive, 10-month long, *classe d’accueil*. Students in these classes are expected to learn French, and to become familiar with Quebecois values and customs, in order to successfully “integrate” into Quebec’s school system and society, more broadly [30]. In this regard, learning French becomes a precursor for engaging in mainstream society [29]. However, young people from refugee backgrounds are often segregated from all other young people in the school they are attending, and remain together, as a group, for all other classes and school activities [29,30]. In this sense, social and linguistic “integration” is questionable. In one study of young people’s experiences in a *classe d’accueil*, participants described how they were strictly forbidden from speaking any language but French, and were offered little individualised support while taking the course, and afterwards, when transitioning to mainstream classes [29].

Although young people may experience different forms of discrimination in this system, schools, nonetheless, appear to be important spaces for young people’s wellbeing. For one, it was mainly through such spaces that young people were able to forge relationships, and connect with others with similar experiences, values and identities:

The first time I reached it it was very, very good for me to talk—to make communication; to make relationships with others.

*(Obed)*

*You don’t feel like, I’m the only person who became this way. Like, there are some other kids who are like me. Yeah, it gives you strength for like—okay […] Yeah, so, it gives you—smile. We get together, we dance, we do whatever. You feel so—not alone*.

*(Jasmine)*

And also in the class there were a lot of immigrants, there were a lot of Colombians. So during the break time, during the lunch time, we would always hang out together. We would speak Spanish, because we could always keep our culture, and we knew that we all have around the same values, the same...the same customs, you understand? [...] It felt good, because at least we could express ourselves. […] we could at least talk to each other. Because it feels bad not to really have any friends in your classes. You feel discouraged, you feel depressed.”

*(Rodrigo)*

These quotes shed light onto the integral role schools play as sites of human connection and belonging. Significantly, school offered participants the opportunity to forge new friendships and to connect through shared values, customs, languages and experiences. As the participants describe, this was particularly important in enhancing their emotional and social wellbeing, while counteracting some of the adverse effects of isolation and cultural invalidation.

Other participants reflected on the importance of key people in their respective school environments:

*When I started school, the principal, she was a support to me... I don’t know how to even describe it; the fact that she believed in and [...] I had teachers that encouraged me—because I was scared, I was scared to, to not be able to integrate and above all I was scared not to be able to succeed*.

*(Leah)*

Participant: My class teacher, and my—I think, Kevin and Melissa. […] I think Melissa and Kevin, they know perfectly. Because they are speak in Nepali also. And it’s not perfect, but they are try to speak in Nepali. My class teacher, Melissa is too much intelligent…[she] speak in Nepali. Yeah, she’s really good […]

Interviewer: So, the fact that she was trying to speak Nepali is one of the things that made you feel safe?

Participant: Yeah.

These accounts demonstrate how young people’s wellbeing and sense of comfort in school environments were also positively influenced by certain individuals in positions of authority, including teachers and principals. In both these testimonies, the participants describe how these people make them feel supported, and even safe in a completely unfamiliar system. The second quote, showing the exchange with the interviewer, sheds light onto the power of connecting with another through language, and the sense of security that this can instil in a young person in this context. Even if the teacher in this situation could not fluently speak the participant’s mother tongue, the sheer effort reflects a form of relating that differs tremendously from more policing and prejudicial styles of relating. This reality stresses the importance of not assuming all formal education, health and social service settings as being inherently hostile. There are people, in all sorts of spaces, that are genuinely caring and that strive to empathize with, and do something about, the difficult positions that these young people find themselves in. These findings echo those identified in studies on young people from refugee backgrounds attending intensive English language schools or centres in Australia [9,13,17]. In these studies, young people remarked on the opportunities these spaces provided in terms of forging relationships with other young people with similar experiences and cultures, as well as in feeling supported and validated by teachers.

The critical consciousness and deep empathy of a few individuals, however, does not redress the participants’ reported experiences of racism and social exclusion. These systemic issues left some participants feeling as though immigrant communities were being stripped of their language, culture and identity. This participant powerfully draws a parallel between cultural assimilation as experienced by Indigenous communities through the residential school system, and the assimilationist processes immigrants are subjected to, including *les classes d’accueil,* in the example of Quebec:

It’s this issue of assimilation that I find not ... really not good. […] The indigenous ... it’s the Europeans really wanted to set up their own system. And they [the Indigenous] were... their children were obligated to go to these [residential] schools. And not speak their language, and they started drinking alcohol. It’s not everyone, but they really have problems now [because of it]. So I tell myself: Immigrants could also lose their identity [like this?], because […] the government controls, I find, they control a lot like what language you learn, what school you go to, what actions you take. If they aren’t okay with the way you act like a good citizen, they’re gonna stop you. So I find they control a lot. Or they say: ‘Oh it’s a free country, free religion” but me I’m not religious, but the law states where your religion could be and what location. And if there are religions that are not too ... that don’t fall in the ‘Western’ category, well they find these...these loopholes to tell you: “No, this, these practices here, don’t you do them.” So it’s really restrictive.”

*(Anna)*

People may argue against this point, emphasizing how the Quebecois are a linguistic and cultural minority in Canada and policies aimed to preserve the French language are integral in protecting the province’s cultural identity. However, before anyone is to set forth an argument defending the assimilationist intent of Quebec’s linguistic and cultural policies, a conversation needs to be had about Quebec’s racially oppressive roots. The fervent call to safeguard Quebecois culture and identity elides the reality that various Onkwehonwe communities were the original people of this land (Onkwehonwe: Haudenosaunee word that in English translates to “the Original People” of this land) [37]. Moreover, it suggests that Quebec was a homogenous society, consisting mostly of white French settlers, when, in actuality, its populace has always been racially, culturally and linguistically diverse. Specifically, the myth of “homogeneity” ignores the central role the slavery of people of colour and Onkwehonwe, and the labour of various migrant communities, including the Chinese and Irish, played in the making of “Quebec” [37,38,39,40]. In this respect, in prioritizing the preservation of one culture, language and identity, the government, and its Quebecois subjects, are effectively endorsing a racial and cultural hierarchy.

The emphasis on cultural assimilation in Quebec’s *classes d’accueil* appears particularly strong in comparison to similar programs in other white-settler nations. For instance, studies on intensive English language schools or centres in Australia reveal the tendency of such programs to adopt a more holistic and sociocultural approach to education and learning [9,13,14,17]. In such environments, young people’s knowledge and values are recognised and celebrated. For instance, many in-class activities centre young people’s identities, customs, and practices through the routine sharing of stories, language, food and festivals [13]. Often, loved ones and family members are welcomed into the classroom, effectively dismantling the school-home divide [13]. Although young people in such environments still report experiences of prejudice and exclusion, and, more generally, continue to contend with inequitable power relations [8,10,11,12,16], the prevailing cultural and racial hierarchy seems to be less rigid and more amenable to intercultural exchange and learning. Consequently, such contexts create powerful opportunities for teachers, and other persons in positions of authority, to connect to, experience and honour the humanity and lived realities of young people from refugee backgrounds. Likewise, by celebrating, rather than erasing, the customs and identities of these students, young people from refugee backgrounds are more likely to feel valued and validated, thereby nurturing the development of more trusting and healing relationships.

## 4. Solutions as Proposed by Participants

In addition to describing their experiences in formal settings, the participants were asked to suggest ways that current systems could be changed to better support young people from war-affected areas who have resettled in Canada. They were also encouraged to conceptualise new services and activities that they felt would better respond to their realities and needs.

Many participants called for institutional changes: Adjustments and improvements to the current systems in place. For instance:

Because [immigrants], to be honest, are more committed about doing something about their lives than anyone else. Because they’ve seen suffering. They see what it’s like to be at the bottom of the barrel. They do not want to make mistakes so that their children suffer like they did. And if the government knows that, they’ll be like, “Shucks, maybe we should invest more in these people because they are the ones were really going to build a foundation. […] it is an investment to be involved in developing those people and not just letting them go and find a job in a factory when they have two or three children to raise, and thinking they will be able to raise their children. Really developing them in the sense that there’s also that whole psychosocial counseling, things like that, that we spoke about, healthcare, education, as well. People who already have some kind of degree and need to get accreditation, Canadian-based, we should be involved in all that in this country.

*(Andy)*

In addition to *seeing* the humanity and immense commitment of immigrants in Canada, and ensuring their access to meaningful opportunities and support, this participant emphasises how people with immigrant and refugee backgrounds should play an active role in supporting the growth of migrant communities and in safeguarding their heritage:

Just because we are asking to be Canadian, doesn’t mean that we want to no longer identify with our own heritage. There is still our own heritage that are holding onto. We still need some of it, you know. We still need that. Perhaps over many generations, everything is going to amalgamate. I don’t know. But right here, right now, I think even for a sense of community, for sense of sanity, it’s not a bad idea to make sure that the government is involved in some of the small community-building, in one way or another.

*(Andy)*

Specifically, in the context of schools, health clinics and hospitals, many participants emphasised the need to recognise and comprehensively address interpersonal and organisational forms of racism, xenophobia, and ignorance, more generally.

*People don’t trust the system. Maybe I don’t trust it. I know a lot of people don’t trust it. People don’t even like to go to the hospital because they know. They don’t have to tell them, “We don’t want you here.” Psychologically, the way they treat you, you feel it. That’s all I can say. If you want me to study social work, bring more people who look like me. So when I talk to people who are like me they will feel comfortable telling me exactly what they’re going through. People relate to people that they see can understand them. If they are Muslim, you talk to me, I don’t have to judge you. I know exactly why you pray. But if you are not, and you’ve never been taught, you lock your mind to what you believe in, it’s not going to work. *

*(David)*

*It would be good if the teachers would be more... more concerned […] if they knew more like for example that there are people that lived through the war in their class, they would pay attention to these people, [and intervene] when comments go too far. Related to the war, related to... for example... the movies that they show us. Yes, maybe it’s too violent. Maybe it’s important to watch, but it’s the comments that come afterwards. We could watch it, but it could be that someone actually lived through that. It’s possible that...instead of reading, instead of finding it funny, to really take it seriously. And […] to know how to respond to students. Like students that say: “Oh me I don’t like immigrants because they will steal our jobs.” The teacher would just sit there, he wouldn’t say anything. To know how to respond like, what is the reality, what are the prejudices. I find there is a gap here for that*.

*(Anna)*

This same participant advocates for programs that foster mutual understanding and connection between young people from refugee backgrounds and those from Quebec and that seek to dispel misconceptions and prejudices that they may hold towards one another.

*Yes for sure that maybe […] the [local] Quebecois students that are from here that...that like this...like being in charge of an immigrant in the beginning to explain the school to them, how it all works. “You have to go here, you have to go there” And that they act as a reference for you maybe. And maybe it becomes a friend. But more so that it is ....so that it has an impact on a wider level, I’m saying, like group gatherings with immigrants and people from here, and talk a bit about what they think about each other. Because for sure there are going to be prejudices on both sides [...] Other resources also. If there are people that are free to listen to us, or that know, that they come to talk to us, that they come to let us know that “We are here” that “We understand that you had to leave your country. If you need anything I am here for you.” Because maybe they have the resources there, but they don’t come and find you. So you don’t know that you are supposed to go look for someone yourself. *

*(Anna)*

The previous quote also highlights the need to have more *active* forms of support. Many of these young people are already struggling enough, and to have to struggle more in order to seek support for the struggles they are already dealing with seems nonsensical. 

Other participants stressed the need to go beyond creating additional programs and adjusting what already exists, and suggested broad-based, transformative changes. These changes focused on Canada’s national image and the hopeful promises it makes, as well as the dominant ways people interact and treat one another.

*The truth has to be told to people. The system has to be honest with people. They give people so much hope that when they come to this country everything changes for them. Oh, your life will be perfect. And people come here, it’s a constant battle. If you want to get help from CLSC you have to fight. If you want to get education, you have to fight. Why? I mean, you are the same people that say we want to accept you in society and making life more difficult for people. Come on. Is that fair? No. The Canadian government, whatever government it is […] has the responsibility to treat people fairly. It’s not a perfect system*.

*(David)*

*And it’s a social issue I think. Because individualism is really strong in rich countries. [And if you don’t live through something really intense with someone] […] there is not that connection, there is not that way of listening [to each other]. So like I said its really in specific places, through things that you go through together, it’s there that one can express themself. When you really know a lot about the person. And I want also to talk about the teachers. The teachers are always not ready for this, and it really doesn’t help either. They aren’t ready… *

*(Anna)*

These accounts highlight how many young people from war-affected areas, over time, come to develop a sense of distrust, isolation and disconnection in a country whose national imagery poorly reflects the often divisive, and individualistic social relations that lie within. Canada is popularly touted as being an open, inclusive, supportive, welcoming and multicultural-loving space [37,38]. However, as described by participants throughout this paper, this does not seem to be an honest portrayal of young people’s experiences in the province’s public institutions, and within society, more generally.

The “taken for granted” legitimacy and inherent benevolence of current systems, and the well-intentioned people who staff them, is another major concern, along with the lack of diversity in these settings. As this participant describes:

But when [people] go in to seek help, [social workers] have to understand where [the person] comes from, what people talk about. It’s not to follow a pre-scheduled way of asking questions without understanding the background. So, for me, it doesn’t work. Refugees come here, go to prison [detention], and then after [detention] you have to work with social worker. You do what you have to do. Because [the social workers] they are part of the system. They follow a rigorous thing, instead of saying, look, maybe was that change this thing. I am not criticizing. I think having social work is a great thing, but there’s something missing. […] The only way social work can change in my opinion is to allow a lot of visible minority people who come from different background to do the work. […] Because the thing is, if I’m talking to you, I already have in my mind, “well, this theory, she’s going to follow the theory and this is the way it works.”

*(David)*

Other participants advocated for the need to create more opportunities and spaces to foster a love of difference and human interconnectivity.

*Yes in this moment we can say, maybe integration activities. Sometimes we see that organizations organize things for people to integrate, and share their culture [with others]. We always see positive things. Because unfortunately, no matter who or no matter what we always see first the negative things in other people. So that’s why people that come are really distrusting of people that are not from the same culture or come from the same country. *

*(Rodrigo)*

This participant insightfully draws attention to the ease with which people see the negative in one another, and from this, make grand generalizations that serve to divide, rather than unite, different communities. The distance such social relations engenders only reinforces the misconceptions different groups have of one another, thereby further fuelling prejudice and human disconnection. This cycle can be broken if intentional spaces are created where people can see the positive in one another and appreciate and honour difference and diversity. This participant provides an example of how such initiatives are already taking shape:

Yes let’s take the example of the family’s house, sometimes they organize... once a year or twice these intercultural activities or we see these dances, these songs from all these countries... Not all but definitely all the countries that the people come from. Do these dances and in there you see integration, you see that there are people interested in understanding new things... a new world that is outside of their own that they live in every day.

*(George)*

## 5. Conclusions

Young people from war-affected countries living in Quebec often find themselves at the intersection of complex forms of oppression that strongly shapes their emotional and mental wellbeing, and their experiences in various formalised spaces, including schools, hospitals and clinics. The hardships endured in their country of origin, compounded by the challenges that come with a new social, cultural, economic and political terrain, can profoundly influence young people’s identity, sense of belonging and experiences of marginality. In other words, it is not strictly, nor necessarily, the violence that they may have experienced in their country of origin that adversely affects these young people’s social, mental, emotional and material wellbeing, but more so the interaction between these past experiences, and the various, and often traumatizing, forms of injustice that many are faced with during and following resettlement.

The degree to which participants’ identities and lived experiences are validated, understood and represented in these spaces may greatly influence how they choose to access and negotiate Quebec’s public health and social services systems. In many cases, the prevailing biomedical model of disease may be out of sync with how participants conceptualise mental health and trauma. Systemic and interpersonal racism, Islamophobia and other forms of prejudice also appear to be central to people’s experiences in these environments, threatening people’s dignity and sense of security.

Participants’ perspectives represented in this study shed light onto the varied difficulties and opportunities young people from war-affected countries negotiate upon resettlement. In particular, the participants’ accounts can raise our awareness of how certain formal spaces of support are experienced as well as enhance our understanding of what is required in creating more inclusive, culturally-responsive, and meaningful services and programs.

By drawing on the framework of intersectionality, it is possible to disentangle the various, and interacting, dynamics that shape these young people’s experiences within certain “formal” spaces. For one, the experiences of war, flight and resettlement are often foreign to those staffing these spaces. Many professionals may not know how to think, feel or act when listening to these young people describe the hardships they have endured. The experiences of flight, migration and resettlement further complicate professionals’ capacity to connect with, and meaningfully support these young people. These realities can be, for many, unimaginable, and overwhelming for professionals. In addition, health and social service professionals are predominantly familiar with approaches to healing, and constructions of mental health, as conceptualized in the Global North, and may have a limited understanding of and appreciation for non-ethnocentric healing modalities [34]. As pointed out by some of the participants, “mental health” is often supported within the family or community, and not seen as an “issue” that warrants outside attention.

In addition to emphasising the power of *lived experience*, intersectionality stresses the need to critically consider how intersecting *marginal identities*, along the lines of race, citizenship status, class, religion and culture, are viewed and treated in these spaces. Experiencing prejudice first-hand, or expecting to experience prejudice, can profoundly affect young people’s sense of security, comfort and belonging in health and social service settings. Being “othered” for wearing a hijab, for being black, and/or for being a migrant can make all the difference in a person’s consideration of accessing, or outright avoiding, these environments.

In other words, when trying to understand young people’s experiences and perceptions of these formal care settings, it is important to consider the *incongruity* between the histories, realities, life perspectives, “healing paradigms”, and identities of these young people, and those of Quebec and Canadian-born professionals. Without striving for greater harmonisation between the norms, values, beliefs and perspectives of these spaces, and the lived experiences and identities of young people affected by war, health and social services risk exposing these young people to structural realities that may perpetuate oppressive and culturally imperialistic dynamics.

Schools also stand to benefit from seriously considering how current teaching methods, and the treatment of students with refugee backgrounds and from war-affected areas, serve to limit young people’s capacity to learn, grow, and develop a sense of belonging. Professionals in these settings need to become more conscious of who is in their classrooms and to explore ways to nurture a learning culture that does not alienate or punish young people from war-affected countries for being who they are. To this end, professionals need to engage in an emotionally rigorous process of self-reflection in order to identify and change their own prejudices and biases against racialised people with refugee backgrounds. Strict segregation of immigrant and Quebec and Canadian-born young people through the *classes d’accueil* in the case of Quebec must also be addressed. This structure only reinforces divisiveness and thwarts opportunities for dialogue, intercultural exchange, belonging and human connection. Adopting a more holistic and sociocultural approach to education, similar to that being used in Australia, may be an important strategy for Quebec in this regard [13,17].

Understanding, through research and community engagement, the complex realities and needs of young people affected by war, and, in particular, their intersectional experiences of oppression, is imperative in order to promote and develop contextually-relevant and inclusive spaces of support in Quebec. Failing to do so will have profound implications for the wellbeing of young people from war-affected areas and their communities, and for Canadian society, as a whole. International migration stemming from war has been increasing globally, and at particularly high levels in Canada [4], and will continue to impact Canadian society. This underscores the importance of reconsidering the rudimentary tenants upon which various spaces of support and learning, namely schools and health care institutions, are built. Importantly, such challenges are not unique to Canada. Rather, they represent contemporary realities that continue to affect and have implications for host countries across the globe. By acknowledging and systemically addressing dominant social relations and structures that reinforce divisiveness as well as cultural and racial hierarchies, it is hoped that young people from war-affected areas will experience less “traumatising” forms of marginalisation, and instead experience more meaningful forms of support, healing and connection.
